# Intradural Extramedullary Spinal Sarcoidosis Mimicking Meningioma

**DOI:** 10.1155/2019/3592980

**Published:** 2019-08-05

**Authors:** Sho Ishiwata, Yoichi Iizuka, Tokue Mieda, Junko Hirato, Hiromi Koshi, Yohei Kakuta, Akira Honda, Hiroyuki Sonoda, Tsuyoshi Tajika, Hirotaka Chikuda

**Affiliations:** ^1^Department of Orthopaedic Surgery, Gunma University Graduate School of Medicine, 3-39-22, Showa, Maebashi, Gunma 371-8511, Japan; ^2^Clinical Department of Pathology, Gunma University Hospital, 3-39-22, Showa, Maebashi, Gunma 371-8511, Japan

## Abstract

**Background:**

Spinal sarcoidosis is a rare subgroup of neurosarcoidosis. Although most sarcoid lesions develop in the intramedullary compartment, intradural extramedullary (IDEM) spinal sarcoidosis is an extremely rare entity.

**Case Presentation:**

We herein report a case of IDEM spinal sarcoidosis mimicking a meningioma. A 32-year-old man presented to the hospital with clumsy hands and was unable to walk without assistance. Magnetic resonance imaging (MRI) of the cervical spine revealed a dural tail sign that is common in meningiomas. The patient underwent gross total resection. The pathological findings consisted with a sarcoid leision of the spinal cord. The patient's myelopathy recovered after surgery.

**Conclusions:**

Physicians should be alert for the possibility of IDEM sarcoidosis mimicking a meningioma in the differential diagnosis of IDEM spinal cord tumors.

## 1. Background

Sarcoidosis is a systemic disease of unknown etiology characterized by noncaseating giant-cell granulomas affecting all organs. Several case series have detected the nervous system involvement of sarcoidosis in up to 5% of all sarcoidosis cases [1]. Furthermore, spinal sarcoidosis, a rare subgroup of neurosarcoidosis, has been noted at clinical presentation in 6% to 8% of cases of neurosarcoidosis [2], although most sarcoid lesions develop in the intramedullary compartment. IDEM spinal sarcoidosis is thus extremely rare, and only 10 cases of IDEM spinal sarcoidosis have been reported [3, 4].

We herein report a case of IDEM spinal sarcoidosis mimicking a meningioma.

## 2. Case Presentation

A 32-year-old man presented to the hospital with progressive numbness and paresthesia of the right hand, trunk, and both lower limbs. He also complained of clumsy hands and was unable to walk without assistance. His history was significant for systemic sarcoidosis two years earlier. Steroid therapy had not been performed because the systemic sarcoidosis was not active, and he had stopped follow-up.

**Figure 1:**
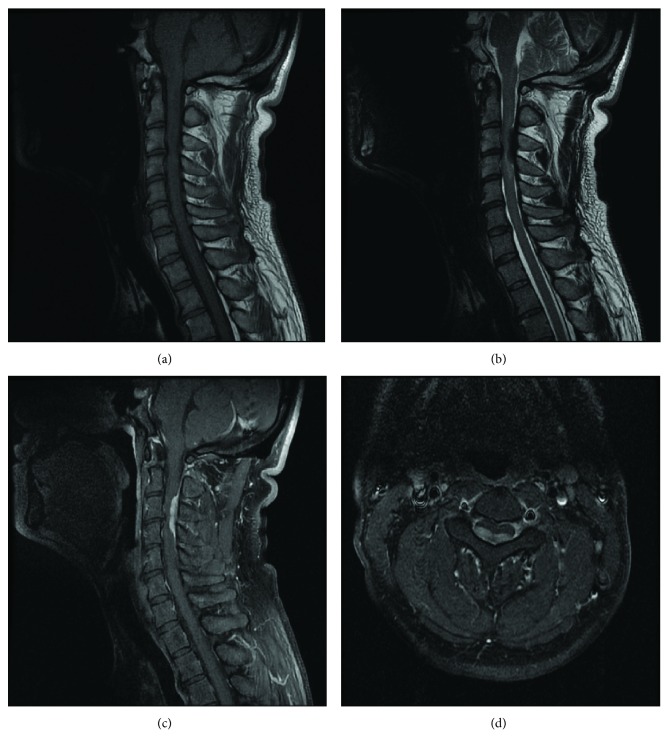
(a, b) MRI of the cervical spine showed IDEM mass, with low intensity in the T2-weighted imaging and isointensity in the T1-weighted imaging at the C3-4 levels. (c, d) The marked enhancement of the mass and a dural tail sign after intravenous admission of Gd-DTPA.

Manual muscle testing was 2/5 in the deltoid, 3/5 in the biceps, 4/5 in the wrist flexors, 5/5 in the wrist extensors, 5/5 in the finger flexors, 4/5 in the finger extensors, 2/5 in the finger abductors, 5/5 in the iliopsoas, 5/5 in the quadriceps, 5/5 in the tibialis anterior, 4/3 in the extensor halluces longus, 5/5 in the gastrocnemius, and 4/5 in the flexor halluces longus. The patient's performance on the 10-second test was slow. Pin prick and temperature sensations were impaired from the T2 dermatome bilaterally. His Japanese Orthopaedic Association (JOA) score for cervical myelopathy, according to the criteria proposed by the association itself, was 3 of 17 points (motor: 0.5/8, sensory: 1.5/6, and bladder and bowel dysfunction: 1/3). Laboratory investigations showed normal values for all parameters. Cervical X-ray and computed tomography (CT) showed no apparent abnormalities. MRI of the cervical spine revealed isointensity in the intradural extramedullary on T1-weighted imaging and a low-intense mass at the C3-4 levels on T2-weighted imaging. Following the intravenous administration of gadolinium-diethylenetriaminepentaacetic acid (Gd-DTPA), an intradural extradural mass with a marked gadolinium enhancement and a dural tail sign were observed (Figure 1).

**Figure 2:**
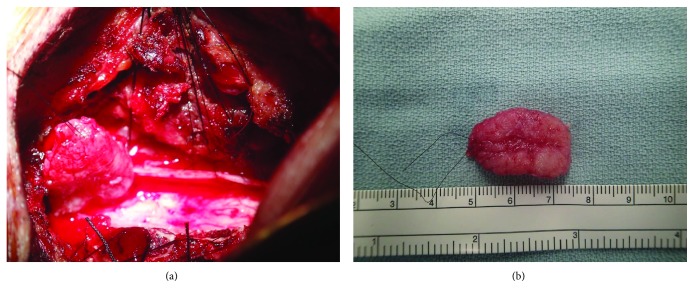
(a) Intraoperative situs of the intradural extramedullary spinal sarcoid lesion. (b) Sarcoid lesion removed by surgery.

**Figure 3:**
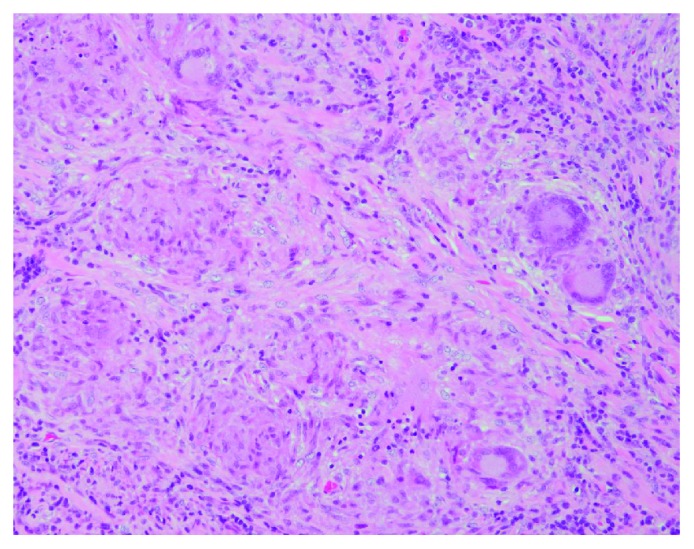
The resected lesion is composed of multiple noncaseating granulomas with fibrosis. Multinucleated giant cells are observed (H&E, original magnification).

The patient underwent laminotomy of the caudal side of the C2, right open door laminoplasty of the C3 to the C4, and laminotomy of the cranial side of the C5. On opening the dura, a firm intradural extramedullary mass was found. The mass was partly adhered to the spinal cord but was not attached to the dura. Of note, the appearance of the actual mass differed from that of a meningioma, as it had been preoperatively suspected to be based on the MRI findings (Figure 2). Gross total resection of the exophytic mass was performed, and the intraoperative histological diagnosis was an epithelioid cell granuloma. Since it had been confirmed not to be a meningioma, resection of the dura was not performed. Histologically, multiple small granulomas were observed in the resected specimen after surgery. Numerous multinucleated giant cells and fibrosis were also seen. While most of the granulomas did not show necrosis, there were some small necrotic foci at the center of the granulomas (Figure 3). Ziehl-Neelsen staining was negative. These pathological findings suggested a sarcoid lesion rather than a tumor or infectious granuloma.

**Figure 4:**
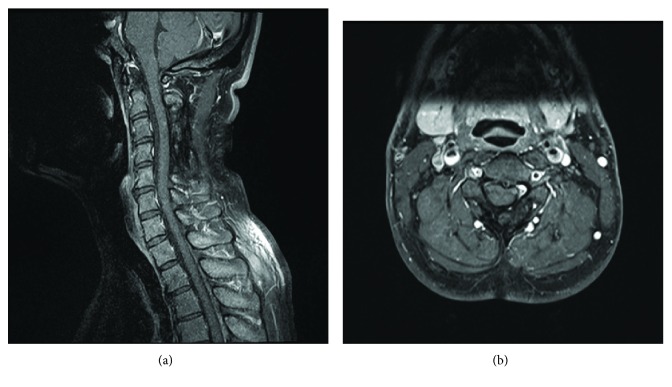
(a, b) MRI of the cervical spine performed four years after the surgery showed no recurrence, and the dural tail sign had disappeared.

After the surgery, the patient's myelopathy recovered gradually and he returned to full-time work. Steroid therapy was not performed because the systemic sarcoidosis was not considered to be active. Four years later, his JOA score for cervical myelopathy was shown to be 14 of 17 points, giving a 78% recovery rate. No abnormalities on MRI were noted at that time (Figure 4).

## 3. Discussion and Conclusions

Spinal sarcoidosis can occur in intramedullary spaces, intraspinal epidural spaces, intradural extramedullary spaces, and vertebral bodies [5]. Most cases of spinal sarcoidosis are intramedullary lesions, and IDEM spinal sarcoidosis is extremely rare [3, 5].

IDEM spinal sarcoidosis has been reported to occur usually in the cervical spine. Indeed, in previous reports, the anatomic location of IDEM spinal sarcoidosis was in the cervical spine in eight cases and thoracic spine in two [2–11]. The typical clinical symptoms of patients with IDEM spinal sarcoidosis include back and leg pain, tingling, numbness or weakness of the extremities, fine motor control deficits, paraparesis, and fecal or urinary incontinence.

Although MRI and CT are useful evaluation tools for diagnosing spinal sarcoidosis, the diagnosis of IDEM spinal sarcoidosis is often difficult because of its similarity to spinal cord tumors. In our case, a mass lesion was observed to be isointense on T1-weighted imaging and low intense on T2-weighted imaging. Although meningiomas usually show iso- or high-intensity findings on T2-weighted imaging, approximately 10% of meningiomas show a low intensity on T2-weighted imaging [12, 13]. In addition, a dural tail sign, which is a relatively characteristic finding of spinal meningiomas, was clearly observed on Gd-DTPA-enhanced MRI. The observed MRI findings were not inconsistent with meningiomas; therefore, it was difficult to diagnose this as a case of spinal sarcoidosis before surgery.

The causes of spinal sarcoidosis remain unclear, although Schaller et al. hypothesized that IDEM spinal sarcoidosis represents a very early stage of intramedullary spinal sarcoidosis [3].

Subtotal or total resection of the mass is required for the surgical treatment of IDEM spinal sarcoidosis. Of note, the risk with surgery in cases of IDEM is lower than that in cases of intramedullary lesions because the mass is extramedullary, so several cases of tumor resection have been reported [3].

There is no consensus regarding the pharmacological treatment of neurosarcoidosis. In previous reports of IDEM spinal sarcoidosis, postoperative steroid treatment was performed in seven cases, but not in three others. Regardless of steroid treatment, recurrence was not observed during the follow-up period [3, 5]. High-dose corticosteroids are recommended for the initial treatment of neurosarcoidosis [2, 4]; however, whether high-dose corticosteroids are appropriate for IDEM spinal sarcoidosis is unclear. In our case, postoperative steroid treatment was not performed, and at four years after surgery, he remained ambulatory without any support and no signs of recurrence on MRI.

Physicians should be alert for the possibility of IDEM sarcoidosis mimicking meningiomas in the differential diagnosis of IDEM spinal cord tumors.

## Abbreviations

IDEM: Intradural extramedullary
MRI: Magnetic resonance imaging
JOA: Japanese Orthopaedic Association
CT: Computed tomography
Gd-DTPA: Gadolinium-diethylenetriaminepentaacetic acid.
